# Andrographolide induces DNA damage in prostate cancer cells

**DOI:** 10.18632/oncotarget.26628

**Published:** 2019-02-01

**Authors:** Ingrid S. Forestier-Román, Andrés López-Rivas, María M. Sánchez-Vázquez, Krizia Rohena-Rivera, Gretchen Nieves-Burgos, Humberto Ortiz-Zuazaga, Carlos A. Torres-Ramos, Magaly Martínez-Ferrer

**Affiliations:** ^1^ Department of Biochemistry, School of Medicine, University of Puerto Rico, San Juan, Puerto Rico, USA; ^2^ University of Puerto Rico Comprehensive Cancer Center, Division of Cancer Biology, San Juan, Puerto Rico, USA; ^3^ Department of Biology, University of Puerto Rico at Rio Piedras, San Juan, Puerto Rico, USA; ^4^ Department of Computer Sciences, University of Puerto Rico at Rio Piedras, San Juan, Puerto Rico, USA; ^5^ Department of Physiology, School of Medicine, University of Puerto Rico, San Juan, Puerto Rico, USA; ^6^ Department of Pharmaceutical Sciences, School of Pharmacy, University of Puerto Rico, San Juan, Puerto Rico, USA

**Keywords:** chemoprevention, phytochemicals, mouse model, DNA repair, gene expression

## Abstract

Prostate cancer (PCa) is the most common diagnosed cancer and is the third cause of cancer mortality in men in the USA. Andrographolide, a diterpenoid lactone isolated from Andrographis paniculata, has shown to possess anticarcinogenic activity in a variety of cancer cells. In this study, we examined the efficacy of Andrographolide in PCa using *in vitro* and *in vivo* models. Androgen-independent (PC3) and androgen-dependent (22RV1) cell lines were treated with Andrographolide to determine the effect in cell motility, cell proliferation and apoptosis. Andrographolide decreased PCa cell migration, decreased invasion, and increased cell apoptosis *in vitro*. Tumor growth was evaluated using an orthotopic xenograft model in which the prostates of SCID mice were injected with 22RV1, and mice were treated three times per week with Andrographolide 10 mg/kg. Andrographolide decreased tumor volume, MMP11 expression and blood vessels formation *in vivo*. Gene expression analysis identified cellular compromise, cell cycle, and “DNA recombination, replication and repair” as the major molecular and cellular functions altered in tumors treated with Andrographolide. Within DNA repair genes we confirmed increased expression of genes involved in DNA double strand break repair. Consistent with this observation we detected increased γH2AX in Andrographolide treated tumors and in cells in culture. Taken together, these data suggest that Andrographolide inhibits PCa by promoting DNA damage.

## INTRODUCTION

Prostate cancer (PCa) is the second most frequently diagnosed cancer in men, with 1.1 million new cases estimated in 2012 and was the fifth leading cause of cancer death in men worldwide in 2012 [1]. Currently, PCa is the most common cancer in men as well as one of the leading causes of cancer-related mortality in the United States [2]. According to the statistics, one out of five men will be diagnosed with prostate cancer [2]. Chemoprevention and prevention with phytochemicals has been widely studied in cancers such as lung, colon, breast and prostate cancer. The combination of natural products with the standard of care treatment is an emerging area of cancer therapeutics with a multitude of benefits such as dose reduction, synergistic effect and delay in development of drug resistance [3–5].

*Andrographis paniculata* is known to possess a variety of pharmacological activities [6–9]. The major component of this plant, Andrographolide, has been reported to have therapeutic potential against liver disorders, common cough and cold, infection, inflammation and cancer in humans [7, 8, 10–15]. For example, Andrographolide has been shown to inhibit cancer cell growth and its 50% growth inhibition ranges from 10 to 28 μM, depending on the type of cancer cell tested which includes the human cancer cell lines SW620 (colon cancer), A498 (renal cancer), NCI/ADR-RES (ovarian cancer), U251 (glioblastoma), HT29 (colorectal cancer), H522 (lung cancer), M14 (melanoma), SKOV3 (ovarian cancer) and DU145 (prostate cancer) [16]. On the other hand, recent reports showed that Andrographolide, at concentrations from 10 to 100 μM, could induce apoptosis in human prostatic adenocarcinoma PC-3 cells and human leukemic HL-60 cells [10, 17, 18]. Previous studies also demonstrate that Andrographolide possesses potent anti-angiogenic activity and, since angiogenesis plays an important role in tumorigenesis, it could have potential therapeutic effects [19, 20].

It has been reported that other phytochemicals, such as curcumin, increase the protein levels of those associated with DNA damage and repair, such as O6-methylguanine-DNA methyltransferase, BRCA1, mediator of DNA damage checkpoint 1, p-p53 and p-H2A.XSer140 in cancer cells, suggesting that this phytochemicals activate a DNA damage response [21, 22]. In this study, we evaluated the role of Andrographolide in prostate cancer using cellular and animal models. We show that Andrographolide decreased prostate cancer cell motility, decreased invasion, and increased apoptosis *in vitro*. In addition, Andrographolide decreased prostate tumor growth, decreased matrix metalloproteinase 11 (MMP11) expression, decreased angiogenesis and altered DNA repair genes such as BRCA2, ATM, BRIP1 *in vivo*. Furthermore, we suggest a possible mechanism of action through which Andrographolide inhibits cancer progression by modulating DNA repair genes. This is, to the best of our knowledge, the first study correlating Andrographolide with double strand DNA repair genes.

## RESULTS

### Andrographolide suppresses proliferation of prostate cancer cells lines

**Figure 1:**
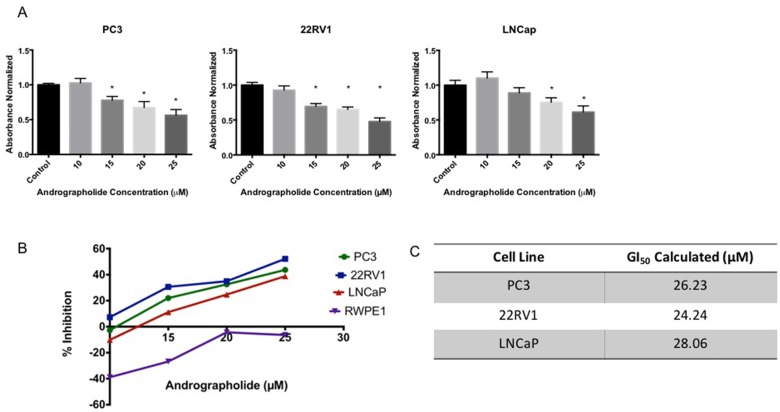
Andrographolide decreased cell viability in prostate cancer cells Cells were incubated with Andrographolide at the indicated doses and cell viability was determined 24 hours or 48 hours after treatment. (**A**) Andrographolide inhibited PC3 cell growth after 48 hours; inhibited 22RV1 and LNCaP cell growth after 24 hours, but did not inhibited RWPE1 cell growth after 24 hours. (**B**) Percentage of cell inhibition was used to determine GI_50_ value. Experiments were made in triplicate. Statistical analysis was performed using one-way ANOVA, followed by Dunnett's test. Mean + SEM (^*^=*P* < 0.05 when compared to control). (**C**) GI_50_ was determined for each cell line.

The anti-proliferative activity of Andrographolide was evaluated using human prostate cancer cell lines (PC3, 22RV1, LNCaP) and normal human prostate cells (RWPE1). Cells were treated with 10–25 μM Andrographolide for 12 hours, 24 hours and 48 hours. Cell viability was measured using the MTS assay. As shown in Figure 1A, Andrographolide inhibited the proliferation of PC3, 22RV1 and LNCaP in a dose-dependent manner. When PC3 cells were treated with Andrographolide for 12 and 24 hours, there were no changes in proliferation (Supplementary Figures 1 and 2). However, Andrographolide inhibited PC3 cell growth at 48 hours of treatment (Figure 1A). 22RV1 and LNCaP cells treated with Andrographolide for 12 hours did not show changes in proliferation (Supplementary Figures 3 and 4). However, Andrographolide inhibited cell proliferation of 22RV1 and LNCaP cells after 24 hours of treatment (Figure 1A). Accordingly, GI_50_ values of Andrographolide were determined for PC3, 22RV1 and LNCap cells as 26.2, 24.2 and 28.1 μM, respectively (Figure 1B, 1C). In order to determine if Andrographolide was toxic to normal cells, we performed MTS Assay using RWPE1 cells. Andrographolide did not inhibit RWPE1 at the tested doses, therefore it is not toxic for normal human prostate cells.

### Andrographolide decreases the migration and invasion of prostate cancer cells

**Figure 2:**
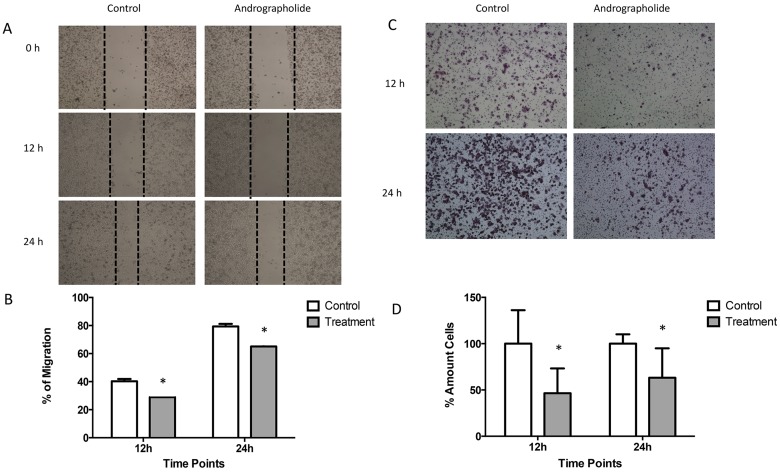
Andrographolide decreased PC3 cell migration and invasion (**A**) Confluent monolayer of PC3 cells was wounded by scratching with a pipette tip and were incubated with or without Andrographolide for 0, 12 and 24 hours. Photomicrographs were taken of PC3 treated with Andrographolide at 0, 12 and 24 hours. (**B**) Quantification of percentage of migration showed that Andrographolide significantly reduced cell migration at 12 and 24 hours when compared to control. (**C**) To evaluate Andrographolide effect in invasion, PC3 cells were incubated for 12 hours and 24 hours with or without Andrographolide. Invasion was evaluated using the boyden chamber method. Photomicrographs were taken of PC3 treated with Andrographolide for 12 hours and 24 hours. (**D**) Andrographolide significantly reduced cell invasion. Experiments were made in triplicate. Statistical analysis was performed using *t*-test. Mean + SEM (^*^P < 0.05).

We investigated the effect of Andrographolide on the migration ability of PC3 cells by using the wound-healing migration assay. For this, a confluent monolayer of PC3 cells were wounded and allowed to migrate for 12 hours and 24 hours (Figure 2A). At 12 and 24 hours, the migration of PC3 cells was significantly reduced by 10% and 15%, respectively, in cells treated with Andrographolide (25 μM) when compared to control (*P* < 0.05) (Figure 2B). PC3 cells treated with Andrographolide for 12 and 24 hours did not show a decreased in proliferation. Thus, the PC3 cells are presenting an inhibition of their migration ability and not due to changes in proliferation. 22RV1 cells were not used for migration assay because they do not grow in a confluent monolayer. Since Andrographolide has been found to inhibit cell invasion in other cancers, we decided to examine the effect of Andrographolide in cell invasion in prostate cancer using androgen-independent PC3. The assay was performed using the Boyden chamber assay for 12 h and 24 h of treatment. Results show that Andrographolide (25 μM) reduced the invasion of PC3 cells by 50 % after 12 hours and by 40% after 24 hours (Figure 2C, 2D). No significant decrease was observed in 22RV1 cell line (Supplementary Figure 5).

### Andrographolide promotes apoptosis in prostate cancer cells

**Figure 3:**
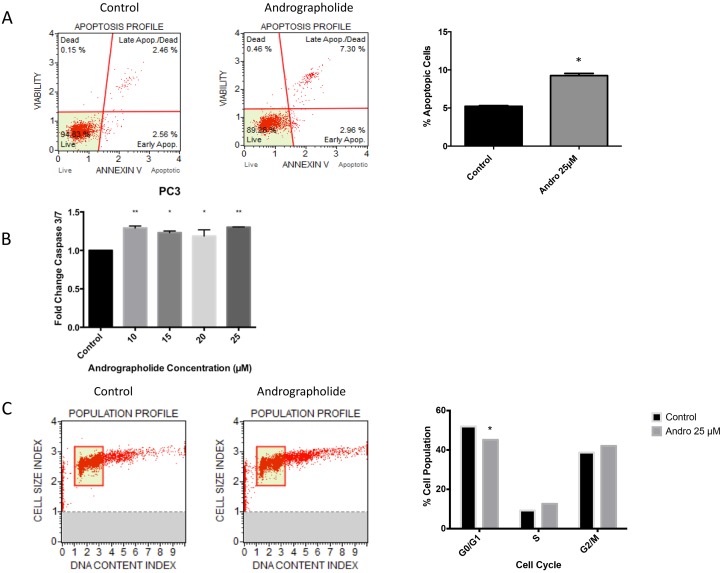
Andrographolide increased apoptosis and decreased cell cycle in PC3 cells PC3 cells were incubated for 24 hours and 48 hours with or without Andrographolide. (**A**) PC3 cells apoptosis was evaluated using flow cytometry (Annexin-V) after 48 hours and measuring Caspase 3/7 activity after 24 hours. Andrographolide significantly increased Annexin-V in PC3 cells when compared to control. (**B**) Andrographolide significantly increased relative activity of Caspase 3/7 in PC3 cell line when compared to control. (**C**) Cell cycle was evaluated using flow cytometry in PC3 cells. Andrographolide (25 μM) significantly reduced cell population at G1/G0 phase and increased cell population at G2/M stage after 48 hours of treatment. Experiments were made in triplicate. Statistical analysis was performed using *t*-test. Mean + SEM (^*^P < 0.05).

To evaluate whether the decrease in cell viability was also accompanied by an increase in apoptosis, we tested whether Andrographolide induces apoptosis in PC3 and 22RV1 prostate cancer cells. PC3 cells were treated with Andrographolide (25 μM) for 24 h and 48 h followed by flow cytometry analysis for Annexin-V. A 50% increase was observed in apoptotic cells after 48 hours of treatment in PC3 cells (Figure 3A). Furthermore, the activity of caspase 3/7 was measured by luminescence in PC3 and 22RV1 cells. After 24 hours of treatment, significantly increased activity of caspase 3/7 was observed in the PC3 cell line (Figure 3B). No significant increase was observed in 22RV1 cell line (Data not shown).

### Andrographolide inhibits cell cycle progression

To evaluate viability and proliferation of PC3 cells, inhibition of cell cycle progression with 25 μM Andrographolide was evaluated at 24 and 48 hours in PC3 cells using flow cytometry. PC3 cells treated with Andrographolide showed a significant decrease in cell number at G1/G0 phase after 48 hours of treatment and an increase of cell population at G2/M phase after 48 hours of treatment. (*P* < 0.05). These results suggest a cell cycle arrest at G2/M phase (Figure 3C). For this assay, 22RV1 cells were not evaluated because they grow in clusters.

### Andrographolide inhibits prostate tumor progression in SCID mice

**Figure 4:**
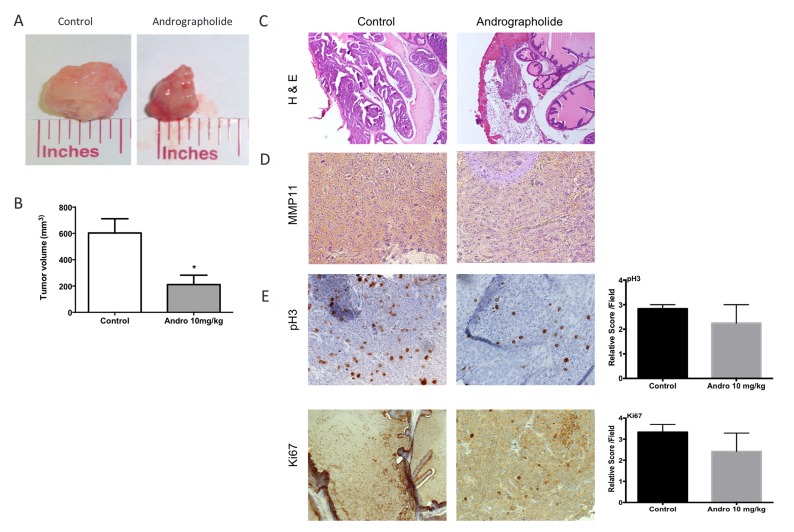
Andrographolide decreased tumor growth, MMP11 expression and proliferation Tumor growth was evaluated using an orthotopic model. Anterior prostate lobes were injected with 22RV1 cells. Tumors were allowed to develop for 4 weeks and Andrographolide was administered three times per week with intraperitoneal injections. The tumor volume was determined with caliper measurements. (**A**) Representative images of tumors show differences in size. (**B**) Tumor volume quantification. N_control_ = 6, N_Andrographolide_ = 6. Mean + SEM (^*^P < 0.05). (**C**) Representative images of vehicle and Andrographolide 10 mg/kg tumors for hematoxylin and eosin (H & E) (**D**) We measured the expression of MMP11 on tumors treated with Andrographolide 10 mg/kg and control. (**E**) The expression of pH3 and Ki-67 was evaluated in 22RV1 tumor tissue. Vehicle at the left; Andrographolide 10 mg/kg at the right. Andrographolide 10 mg/kg reduced cell proliferation *in vivo*. Statistical analysis was performed using *t*-test. Mean + SEM (^*^P < 0.05).

To test the effect of Andrographolide on prostate tumor progression, a SCID orthotopic model was used to develop tumors in the anterior prostate lobes using 22RV1 cells. One week following cell injection, mice were treated 3× per week, by intraperitoneal injections, with vehicle or 10 mg/kg Andrographolide for four weeks. Mice treated with Andrographolide 10 mg/kg developed smaller tumors when compared to control tumors. The tumors treated with Andrographolide 10 mg/kg had three times lower tumor volume than control tumors (Figure 4A, 4B). Pathological, histological and immunohistochemical analyses of collected tumor tissue were used to study the effect of Andrographolide in tumor biology. Slides were examined by a pathologist at low, medium and high power under a compound light microscope. Tumor assessment was made as described by Isaacs and Hukku [23]. Tumors were classified in four categories by degree of differentiation: well differentiated, moderately differentiated, poorly differentiated, and anaplastic. Well differentiated tumors are characterized by the presence of glandular structures, lumen, basement membrane, and stroma. Moderately differentiated tumors are characterized by smaller glandular structures with the lumen obstructed by tumor cells. However, the basement membrane and stroma remained intact. Tumors classified as poorly differentiated have absence of glandular structures, basement membrane, and do not show a consistent relationship between tumor cells and stroma. Individual tumor cells, however, still show a normal nucleus to cytoplasm ratio. Tumors classified as anaplastic lack appearance of tissue organization and individual tumor cells show irregular nucleus size and abnormal nucleus to cytoplasm ratio. All tumor samples, regardless of the treatment, were classified as histologically anaplastic showing no significant differences among treatments (Figure 4C).

### Andrographolide decreases MMP11 expression proliferation and blood vessel formation *in vivo*

**Figure 5:**
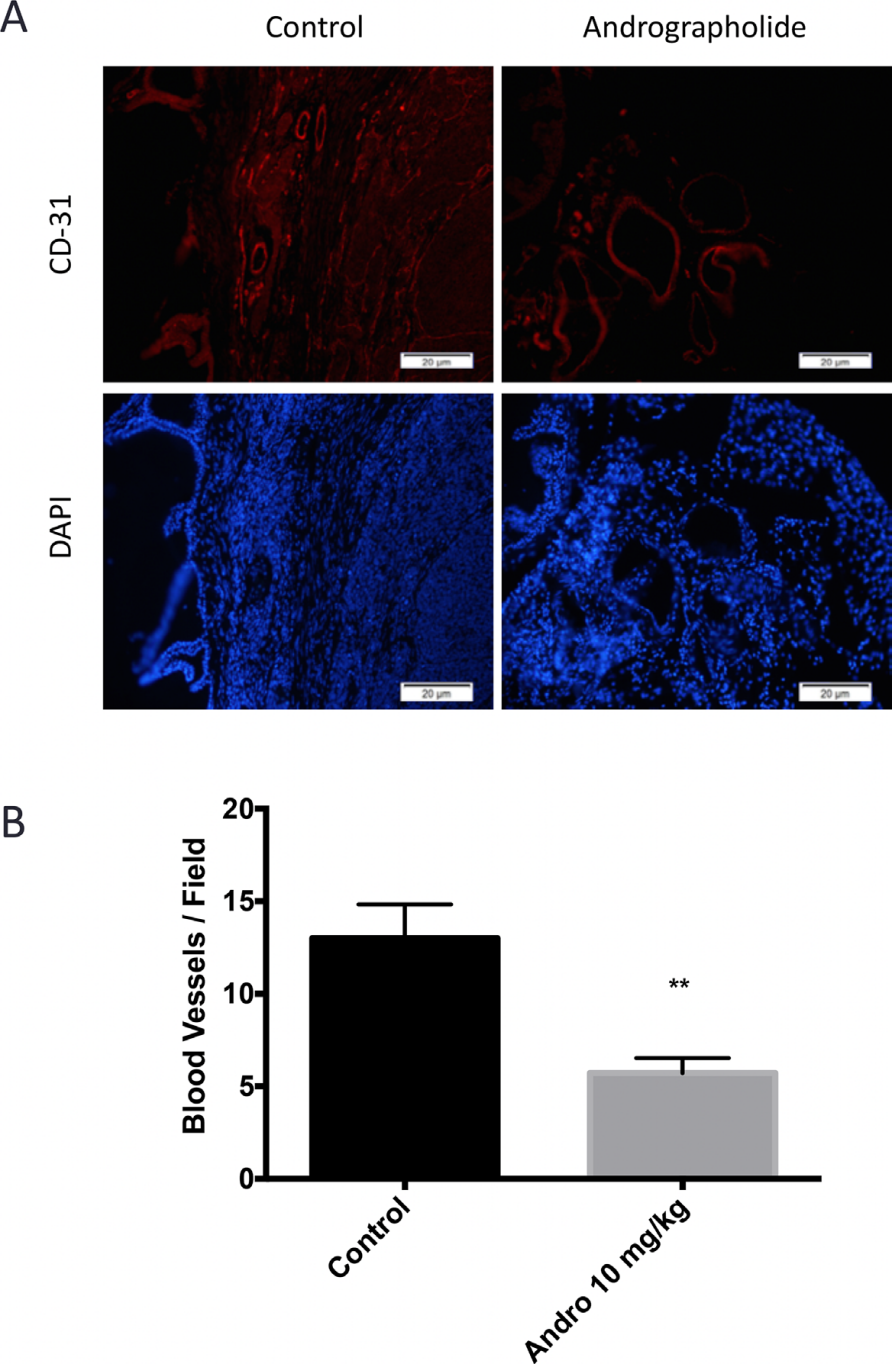
Andrographolide decreased CD31 expression (**A**) The expression of CD31 was evaluated in 22RV1 tumor tissue. Vehicle at the left; Andrographolide 10 mg/kg at the right. Nuclei are stained in blue and blood vessels are stained in red. (**B**) Andrographolide significantly reduced CD31 expression. Experiments were made in triplicate. Statistical analysis was performed using *t*-test. Mean + SEM (^*^P < 0.05).

The effect of Andrographolide on MMP11 was evaluated to determine if the expression of this protein, involved in the breakdown of extracellular matrix, was also affected by Andrographolide. For this, we performed an immunohistochemical assay. We found a decrease in MMP11 expression in treated tumors (Andrographolide 10 mg/kg) when compared to control (Figure 4D). The expression of the proliferation markers pH3 and Ki-67 were evaluated to determine the effect of Andrographolide in this cancer hallmark. In this study, we found that Andrographolide decreased the expression of pH3 and Ki-67 in mice tumors when compared to control (Figure 4E). Additionally, one of the cancer hallmarks, angiogenesis, is essential to cancer progression since the formation of new blood vessels helps tumors to grow. To test the effect of Andrographolide in the formation of blood vessels, the expression of CD31 was measured by immunofluorescence. In our study decreased formation of blood vessels in tumors from mice treated with 10 mg/kg Andrographolide was observed compared to those treated with control (Figure 5).

### Andrographolide alters genes associated with cellular compromise, cell cycle, and DNA recombination, replication and repair

**Figure 6:**
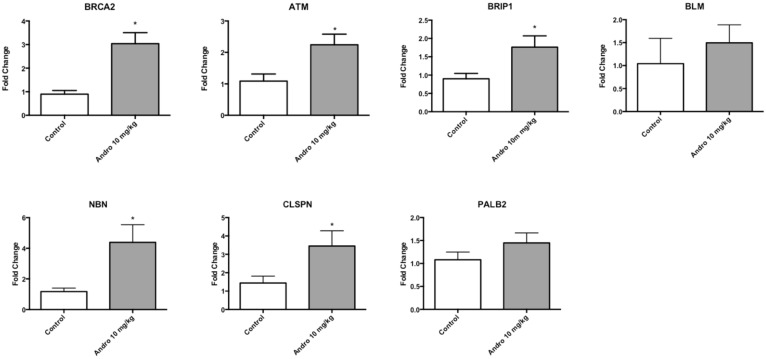
Andrographolide 10 mg/kg altered genes associated with DNA repair Real time PCR results for: ATM serine/threonine kinase (ATM), Bloom syndrome, RecQ helicase like (BLM), Breast cancer 2, early onset (BRCA2), BRCA1 interacting protein C-terminal helicase 1(BRIP1), Nibrin (NBN), partner and localizer of BRCA2 (PALB2). Experiments were made in triplicate. Statistical analysis was performed using *t*-test. Mean + SEM (^*^P < 0.05).

Microarray analysis was performed using mice tumor samples to identify the effect of Andrographolide 10 mg/kg on gene expression. Andrographolide 10 mg/kg treatment significantly altered the expression of 675 genes classified in three broad molecular and cellular functions: cellular compromise, cell cycle, and “DNA recombination, replication and repair”. Among the DNA recombination, replication and repair category, the following genes were represented: ATM serine/threonine kinase (ATM), Bloom syndrome, RecQ helicase like (BLM), Breast cancer 2, early onset (BRCA2), BRCA1 interacting protein C-terminal helicase 1(BRIP1), Claspin (CLSPN), Nibrin (NBN), and partner and localizer of BRCA2 (PALB2) (Table 1). A common feature of these genes is that they are associated with the double-strand break repair pathway. To validate that the expression of this group of genes is affected by Andrographolide, we performed real time PCR assays. We observed an increased in the expression of double strand break repairs genes: ATM serine/threonine kinase (ATM), Bloom syndrome, RecQ helicase like (BLM), Breast cancer 2, early onset (BRCA2), BRCA1 interacting protein C-terminal helicase 1(BRIP1), Nibrin (NBN), partner and localizer of BRCA2 (PALB2) (Table 1). Andrographolide significantly increased the expression of ATM, BRCA2, BRIP1, CLSPN, and NBN. Also, an increase in BLM and PALB2 genes expression was also observed in tumors treated with Andrographolide. (Figure 6). The primers used for real time PCR confirmation are listed in Table 2.

**Table 1:** Microarray analysis: Genes associated with cell cycle and DNA repair altered by Andrographolide *in vivo*

Gene symbol	Description	Fold change	*P* value	qPCR-*P* value
*ATM*	ATM serine/threonine kinase	2.14	3.07E-02	2.15E-02
*BLM*	Bloom syndrome, RecQ helicase-like	2.49	3.15E-02	3.21E-01
*BRCA2*	Breast cancer 2, early onset	2.23	3.90E-02	1.50E-03
*BRIP1*	BRCA1 interacting protein C-terminal helicase 1	2.08	4.35E-02	2.30E-02
*CLSPN*	Claspin	2.06	2.91E-02	4.28E-02
*NBN*	Nibrin	2.18	2.94E-02	2.10E-02
*PALB2*	partner and localizer of BRCA2	2.99	2.47E-03	2.04E-01

**Table 2:** Primer sequences for qRTPCR analysis of genes affected by Andrographolide *in vivo*

Primer	Sense	Antisense
BRCA2	5′-GAAGCCCTTTGAGAGTGGAA-3′	5′-CTCCATCTGGGCTCCATTTAG-3′
ATM	5′-TGCACTGAAAGAGGATCGTAAA-3′	5′-CAGAGGGAACAAAGTCGGAATA-3′
NBN	5′-CCACACATCATTGGAGGATCA-3′	5′-GTACCTCCATTTCCTGCCTTAG-3′
BRIP1	5′-CAGCTGGAGGCTAATCATATC-3′	5′-CTGGAAGGTAGCACAGAGATTC-3′
CLSPN	5′-GGAGGAGGAATTTGGAGACTTT-3′	5′-TCTTCCAGTGCCAGATCATTAC-3′
BLM	5′-CAGGATGGCTGTCAGGTTATC-3′	5′-GGTAGTAACCCTCCACAGATTTAG-3′
PALB2	5′-CTGGAAGGTGACGTGAAAGA-3′	5′-CAGTACACTGACCGAGAAGTAAG-3′

### Andrographolide increases histone H2AX phosphorylation in prostate cancer cells

**Figure 7:**
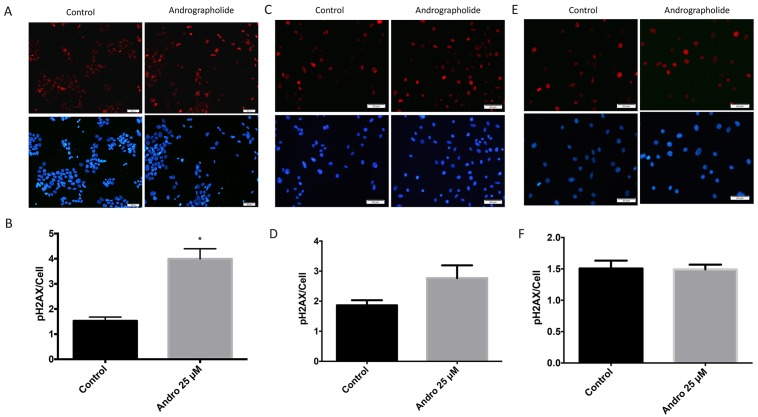
Andrographolide increased expression of phospho-H2AX histone in prostate cancer cells To evaluate the effect of Andrographolide in phospho-H2AX, a double strand DNA break marker, 22RV1, PC3 and normal prostate cells were treated with 25 μM of Andrographolide and incubated for 24 hours. (**A**) Representatives images of phospho-H2AX staining for 22RV1 cells treated with Andrographolide. Vehicle (left panel); Andrographolide 25 μM (right panel). Nuclei are stained with DAPI (blue) and phospho-H2AX is stained in red. (**B**) Statistical analysis shows that phospho-H2AX was significantly increased in 22RV1 cells when treated with Andrographolide. (**C**) Representatives images of phospho-H2AX staining for PC3 cells treated with Andrographolide. Vehicle (left panel); Andrographolide 25 μM (right panel). Nuclei are stained with DAPI (blue) and phospho-H2AX is stained in red. (**D**) Statistical analysis shows that phospho-H2AX was increased in PC3 cells when treated with Andrographolide. (**E**) Representatives images of phospho-H2AX staining for normal prostate (RWPE1) cells treated with Andrographolide. Vehicle (left panel); Andrographolide 25 μM (right panel). Nuclei are stained with DAPI (blue) and phospho-H2AX is stained in red. (**F**) Statistical analysis shows that phospho-H2AX levels did not change in RWPE1 cells when treated with Andrographolide. Experiments were made in triplicate. Statistical analysis was performed using *t*-test. Mean + SEM (^*^P < 0.05).

Immunofluorescence studies were performed in 22RV1, PC3 and normal (RWPE1) cells to determine the effect of Andrographolide in the levels of phosphorylated histone H2AX (γH2AX), a marker of DNA double-strand breaks [24]. We found that, 24 hours after of Andrographolide treatment (25 μM), there was a significant 1.6-fold increase in γH2AX in 22RV1 cell line (Figure 7A, 7B). These results indicate that Andrographolide induces DNA double-strand breaks. For PC3 cells we found that there was a 0.6-fold increase in γH2AX when treated with Andrographolide for 24 hours (Figure 7C, 7D). For normal prostate cells, we found that Andrographolide has no effect in the levels of γH2AX (Figure 7E, 7F).

### Andrographolide induces DNA Damage in PC3 cells

**Figure 8:**
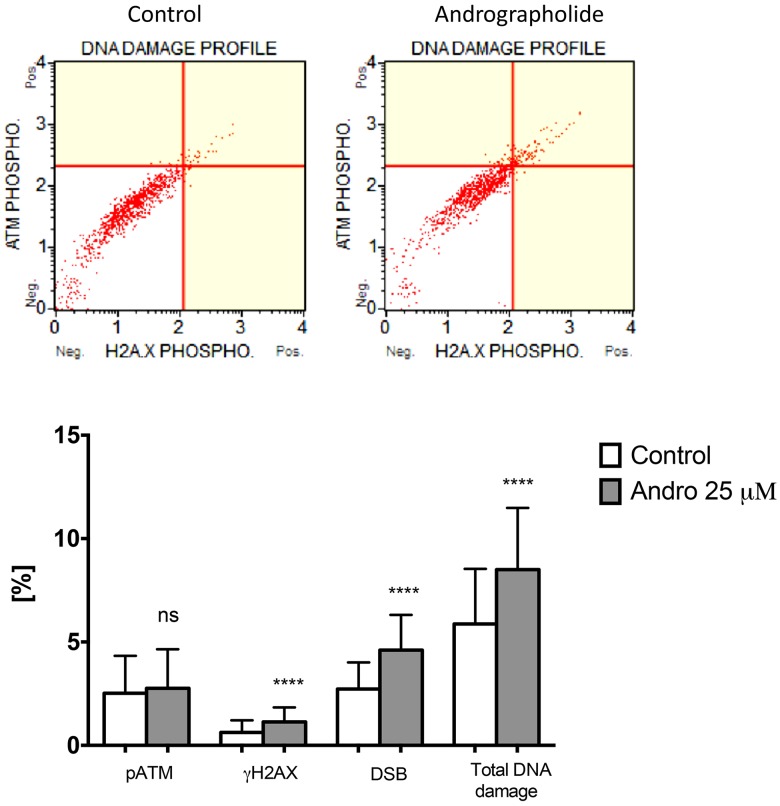
Andrographolide increases DNA damage in PC3 cells Cells were incubated with Andrographolide 25 μM or vehicle (Control) for 24 hours. DNA damage was determined with a flow cytometry approach that detects cells that simultaneously express phosphorylated ATM and γH2AX. DNA damage is presented relative to Control cells, which is set at 1. Experiments were made in triplicate. Statistical analysis was performed using *t*-test. Mean ± SEM (^*^P < 0.001).

To further evaluate the presence of DNA damage in Andrographolide treated cells, we used a flow cytometry method that detects phosphorylated ATM and γH2AX. DNA damage is expressed as the percent of cells that simultaneously display both markers. PC3 cells treated with 25 μM Andrographolide for 24 hours show a significant increase in DNA damage as compared to control cells (Figure 8). For this assay, 22RV1 cells were no evaluated because they grow in clusters and cannot be processed by the flow cytometer.

## DISCUSSION

Andrographolide, whose major component is diterpene lactone, has been shown to possess antitumor properties. However, its mechanism of action is not fully understood. It has been observed that Andrographolide inhibits cancer cell proliferation, induces cell-cycle arrest, and promotes apoptosis [18, 25, 26]. Previous studies showed the potential of Andrographolide as a chemopreventive agent by suppressing growth of cancer cells by inhibiting NF-κB, PI3K/AKT and other kinase pathways and by inducing apoptosis [8, 27]. In prostate cancer, Andrographolide has been known to inhibit cell viability and cell migration by modulating CXCL11, CXCR3, CXCR7, and IL-16 expression [28, 29]. Nevertheless, the role of Andrographolide in PCa progression has not been elucidated.

In this work, we evaluated the effects of Andrographolide in PCa using *in vitro* and *in vivo* models. We focused on cell migration, invasion, tumor growth, proliferation, angiogenesis, changes in gene expression and DNA repair. We assessed the efficacy of Andrographolide *in vitro* using PC3, 22RV1 LNCaP PCa cell lines and RWPE1 prostate cells. Andrographolide exhibits anti-proliferative activity on these cells in a dose-dependent manner. LNCaP are less sensitive to this compound, as compared to PC3 and 22RV1 cells. Thus, Andrographolide has potential to target PCa cells that are aggressive in nature. In contrast, the same doses did not inhibit the proliferation of RWPE1, a cell line derived from normal human prostate cells. We observed that cell population remained constant at higher doses. This strongly suggests that Andrographolide is not toxic for normal prostate cells. The resistance of noncancerous cells to Andrographolide will require additional study but this characteristic strengthens the rationale the use of Andrographolide as a chemotherapeutic agent. We observed that Andrographolide inhibited cancer cell motility and invasion in prostate cancer cells. Further, results show that Andrographolide induces cellular apoptosis via changes in caspase 3/7 activity. Previous studies have shown that the protein TRAIL is capable of initiating apoptosis through the activation of caspases-3 and -7 [30, 31]. Our results show that Andrographolide can restrain proliferation and stimulate apoptosis in aggressive prostate cancer cells specifically in androgen insensitive (AI) cell (PC3) since cancer cells that undergoes androgen receptor (AR) activation can cause proliferation and block apoptosis [32]. Additionally, flow cytometry with Annexin V staining was conducted to quantify apoptotic cells. An increase of apoptotic cells was observed in PC3-treated cells. This result further validated that Andrographolide could inhibit the growth of prostate cancer cells and subsequently induce apoptosis.

DNA damage is a molecular event which is closely associated with cell cycle arrest and apoptosis. To date, several studies showed that Andrographolide effectively induces cell cycle arrest at G0/G1 stage in most of the cancer cells [10, 33]. Our findings show that Andrographolide decreased cell population at G0/G1 stage but increased cell population at G2/M phase. This arrest may be due to different cyclin-dependent kinase (CDKs) activities being regulated with the association of their cyclin partners, kinases, phosphatases and specific inhibitors [34]. The deregulation of cyclins (D & E) and cdks (2,4 & 6) can results in abnormal growth of cells since they play important roles in the progression of cells through the G_0_/G_1_ phase of the cell cycle [35]. Therefore, further research is needed to examine the detail mechanism of cell cycle arrest in Andrographolide-treated PC3 prostate cancer cells.

Given that Andrographolide has been shown to decreased breast, renal, melanoma and lung tumor volume [36–39], we performed *in vivo* studies. Our data *in vivo* showed a significant decreased in tumors treated with Andrographolide. Tumor differentiation, invasion and metastasis are controlled by multiple factors during the multistep processes of extracellular matrix (ECM) composition and degradation [40]. In addition, new tumor blood vessels secrete various proteolytic enzymes which further add to the complex picture of tumor progression. Since matrix metalloproteinase 11 (MMP11) belongs to a group of zinc-dependent proteases involved in ECM and basement membrane degradation, we measured its expression in tumors treated with Andrographolide [41, 42]. MMPs promote the formation of new blood vessels, and are involved in numerous processes associated with tumor cell growth, differentiation, invasion, diffusion and metastasis [41, 42]. Our results show that Andrographolide decreases the expression of MMP11 in tumor tissue. Furthermore, our results show that Andrographolide decreases the number of blood vessels, confirming the observed inhibition in tumor growth. The contribution of MMPs to angiogenesis is not only by the degradation of basement membrane that cause endothelial cells to detach and migrate into new tissue, but also by releasing proangiogenic factors, bound to ECM, such as bFGF, VEGF, and TGFβ [43].

Additionally, we measured if Andrographolide inhibited profileration *in vivo*. We observed that Andrographolide 10 mg/kg decreased pH3 and Ki-67 expression in tumor tissue. These findings are important since proliferation is one of the cancer hallmarks. One of the main mutation in prostate cancer includes PTEN gene. It is known that loss of function in PTEN amplify PI3K signaling and promote tumorigenesis in a variety of experimental models of cancer. Although we found that Andrographolide arrests cell cycle at G2/M phase, more studies will need to be conducted to understand the specific mechanism whereby Andrographolide is decreasing these two proliferation markers.

Our microarray and real time PCR experiments indicate that Andrographolide induces the expression of genes associated with double strand break DNA repair in tumors. The expression of ATM serine/threonine kinase (ATM), Bloom syndrome, RecQ helicase like (BLM), Breast cancer 2, early onset (BRCA2), BRCA1 interacting protein C-terminal helicase 1(BRIP1), Nibrin (NBN), partner and localizer of BRCA2 (PALB2) was increased in tumors treated with Andrographolide 10 mg/kg.

It has been suggested that BRIP1 may have an anti-oncogenic role, and its downregulation has been observed in multiple types of cancer [44]. Given that BRIP1 has an essential function in the regulation of normal cell cycle progression and DNA repair, the BRIP1 gene represents a good candidate for prediction of genetic susceptibility to cancer [45–48]. Our results show that Andrographolide increased the expression of BRIP1. BRCA2 plays a limited role in DNA recombination and repair processes, but seems to be a key player in the regulation of the RAD51 recombinase activity, a protein involved in repairing double stranded DNA breaks [49–51]. We showed that Andrographolide increased the expression of BRCA2 gene. Furthermore, our results show that Andrographolide increased the expression of ataxia-telangiectasia mutated (ATM) in prostate tumors. ATM is a serine/threonine protein kinase belonging to the PI3K family and it functions as a key mediator of DNA damage response and induces cell cycle arrest [52].

NBN function in combination with RAD50 and MRE11 proteins to form the MRN complex, responsible for the detection of DSBs and the subsequent activation of repair mechanisms [53]. Consequently, NBN is expected to act as a tumor suppressor gene by recruiting ATM [53]. Interestingly, this gene was demonstrated to be increased by Andrographolide in tumors. On the other hand, it is well known that BLM mutations lead to Bloom Syndrome, a disease characterized by growth retardation and predisposition to cancer [54, 55]. Therefore, an increased expression of this gene will be essential to reduced cancer predisposition. We showed that Andrographolide increased the expression of BLM gene. PALB2 was found as a tumor suppressor gene [56]. Accordingly, we found that Andrographolide increased the expression of this tumor suppressor.

We determined the phosphorylation status of the H2AX histone (γH2AX) to confirmed an increase in double strand breaks since γH2AX functions as an early indicator of DNA double-strand breaks (DSB) and of the resulting response (DNA damage response [DDR]) [24, 57]. Histone H2AX is a substrate of several phosphoinositide 3-kinase-related protein kinases (PIKKs), such as ATM, which is considered a major physiological mediator of H2AX phosphorylation in response to DSB formation [58]. We found that Andrographolide significantly increased γH2AX in 22RV1 cells. This finding is in accordance with the increased expression of double-strand break DNA repair genes after Andrographolide that we report. Furthermore, using a method that simultaneously detects phosphorylated ATM and γH2AX we observed that Andrographolide induces DNA damage in PC3 cells. ATM is known to phosphorylate checkpoint kinase 2 (CHK2) which in turn phosphorylates the tumor suppressor p53, disrupting its binding to its negative regulator MDM2. This results in p53 protein stabilization and allows p53 to mediate its downstream effect on cell growth suppression [59, 60]. Consequently, the activation of the above-mentioned cell cycle checkpoints can result in suppression of cell growth and activation of apoptosis. It has been observed that the functional status of p53 is important in the progression of prostate cancer and its response to treatment. Androgen-dependent cells, 22RV1 are known to have the presence of wild type p53, while androgen-independent, PC3 cells are known to be p53 null [61]. This can explain our findings where Andrographolide decreased PC3 cell proliferation after 48 hours but after 24 hours for 22RV1 line.

In summary, we evaluated several hallmarks of cancer like proliferation, cell motility, tumor growth, angiogenesis, and changes at the genetic level. Importantly, we observed that Andrographolide increased genes associated with double strand DNA repair. This increase may explain the mechanism whereby Andrographolide induces a DNA damage response involving cell growth suppression, cell cycle arrest and apoptosis. Specifically, DNA double strand break-dependent ATM activation may result in p53 activation, which result in apoptosis and arrest of cell cycle. Taken together, we have shown that Andrographolide is an effective anti-cancer compound for prostate cancer by regulating genes associated to double-strand breaks such as ATM, NBN, BRCA2 BLM, PALB2 and BLM. This innovative finding is essential to understand the mechanism of action of Andrographolide in prostate cancer. Further studies are needed to delineate the mechanisms by which Andrographolide suppresses prostate cancer progression *in vivo*.

## MATERIALS AND METHODS

### Cell culture

Human androgen-independent prostate cancer cells PC3, human androgen-dependent prostate cancer cells 22RV1 and LNCaP, and human normal prostate cells RWPE-1 were purchased from American Type Culture Collection (ATCC), (Manassas, VA, USA). The PC3, 22RV1 and LNCaP cell lines were maintained in RPMI-1640 medium with L-glutamine (Hyclone, MA, USA) supplemented with 10% fetal bovine serum (FBS), 100 U/mL penicillin and 100 μg/mL streptomycin (Gibco, Carlsbad, CA, USA). RWPE1 cells were maintained in Keratinocyte-SFM supplemented with L-glutamine, 0.05 mg/mL of Bovine Pituitary Extract (BPE) and 5 ng/mL of Epidermal Growth Factor (EGF) (Gibco, NY, USA). Cells were maintained in a 5% CO_2_ humidified atmosphere at 37° C.

### Andrographolide solution

Andrographolide was obtained from Sigma-Aldrich (Saint Louis, MO, USA), dissolved in dimethyl sulfoxide (DMSO) at a concentration of 100 mM, and then diluted with complete growth medium to the final desired concentration. Samples treated with DMSO alone were used as vehicle.

### Cell viability

Cell viability and proliferation were assessed using CellTiter 96^®^ AQ_ueous_ One Solution Cell Proliferation Assay (MTS) (Promega, Madison, WI, USA) and was used according to the manufacturer's instructions. A total of 10,000 cells per well of PC3, 22RV1, LNCaP and RWPE1 were grown in a 96-multiwell plate. After 24 hours of incubation cells were treated with DMSO (control) and Andrographolide at concentrations of 10, 15, 20 and 25 μM in fresh complete medium. MTS reagent was added at 24 and 48 hours of treatment and incubated for 2 hours. Absorbance was measured at 490 nm using a xMark Microplate Absorbance Spectrophotometer (BIORAD, USA). GI_50_ was determined by calculating percent of inhibition as [(C-T)/C] × 100 (C = control; T = treatment) and using the slope equation y = mx + b where y = 50. Experiment was carried out in triplicates and data was analyzed using GraphPad Prism ANOVA followed by Dunnett's comparison at 95% confidence interval.

### Cell migration assay

Cell migration was assessed using the scratch (wound healing) method. PC3 cells were grown in 6–well tissue culture plates until 95% confluency. A scratch was made through the confluent monolayer using a sterile plastic 200 μL pipette tip. Cells were gently washed with PBS and incubated at 37° C in RPMI-1640 supplemented with 10% fetal bovine serum in the presence of vehicle (DMSO) or Andrographolide (25 μM). Cells were photographed using a Nikon Eclipse TS100 microscope (Nikon, Tokyo, Japan) at 12 and 24 hours of treatment at a 4× magnification. A total of 10 distance measurements within each wound were obtained and analyzed using Image Pro Plus Software. The differences in wound closure were normalized to 0 hours according to the treatment and compared to the control using Student's *T*-test at a 95% confidence interval. All experiments were performed in triplicate.

### Cell invasion assay

PC3 and 22RV1 cells (4 × 10^4^/well) were seeded in the upper chamber of a transwell system (Corning Incorporated, NY, USA) in serum-free medium, with or without Andrographolide (25 μM) and invasion assay was carried out according to the manufacturer's instructions. Serum-containing medium was added to the lower chamber to act as a chemotactic attractant. At 12 and 24 hours of incubation, the upper chamber was removed and unmigrated cells were removed with PBS. Cell that migrated across the membrane were fixed with 10% formalin, stained with hematoxylin overnight, washed with deionized water, and these membranes were mounted on slides. Photographs at a 4× magnification were captured with a Nikon Eclipse TS100 microscope (Nikon, Tokyo, Japan). The number of invasive cells was counted using Image Pro Plus Software. Results were analyzed using the Student's *T*-test at a 95% confidence interval. All experiments were performed in triplicate.

### Flow cytometry

Cell cycle, apoptosis and DNA damage assays were performed using flow cytometry with the Muse^®^ Cell Analyzer instrument (EMD Millipore Merck KGaA, Darmstadt, Germany). Cell cycle was assayed by incubating PC3 cells (2.5 × 10^6^ cells/mL) with DMSO (control) or Andrographolide (25 μM) for 24 and 48 hours and cells (detached and attached) were collected and fixed with ethanol 70%. Muse^®^ Cell Cycle Assay Kit was used following manufacturer's instructions. Annexin-V was used to determined apoptosis in PC3 cell line. Cells (1× 10^7^ cells/mL) were incubated with DMSO (control) or Andrographolide for 24 and 48 hours and all cells (detached and attached) were stained using Muse^®^ Annexin-V Kit following manufacturer's instructions. DNA damage was assayed by incubating PC3 cells with DMSO or Andrographolide 25 μM for 24 hours and cells were collected, fixed and permeabilized. Cells were evaluated using Muse™ Multi-Color DNA Damage Kit following manufacturer's instructions. The experiments were done in triplicates and data were analyzed using GraphPad Prism Student *t*-test at 95% confidence interval.

### Apoptosis assay

Caspase 3/7 activity was measured to evaluate apoptosis in PC3 and 22RV1 cell lines. A total of 10,000 cells per well were seeded in a 96-white walled multiwell plate. After 24 hours of incubation, cells were treated with DMSO (control) and Andrographolide (10, 15, 20 and 25 μM) in fresh complete medium. Following treatment, Caspase-Glo 3/7 reagent was added and incubated at room temperature for 60min. Caspase-3/7 activities were determined by quantifying luminescence. Experiments were performed in triplicates and data were analyzed using GraphPad Prism ANOVA followed by Dunnett's comparison at 95% confidence interval.

### Orthotopic mouse model

All animal studies were carried out in accordance to the Institutional Animal Care and Use Committee regulations. Seven to eight weeks male ICR-SCID mice (Taconic, Germantown, NY, USA) were housed and maintained in a pathogen-free animal facility. An orthotopic xenograft model in which 22RV1 (250,000 cells) were injected in the anterior prostate lobes was used. Cells suspended in PBS were placed in 30 μL of collagen I (Becton Dickinson, Franklin Lakes, NJ, USA), allowed to slightly solidify and injected in the anterior prostate lobes. Mice were intraperitoneally injected three times per week with Andrographolide (10 mg/kg) or vehicle (saline). After 4 weeks of treatment, tumors were collected, measured, and fixed in 10% buffered formalin or snapped frozen.

### Hematoxylin-eosin staining

Paraffin-embedded tumor tissue sections (5 μm) were deparaffinized in xylene and hydrated using serial descending concentrations of alcohol. Tissue was stained with hematoxylin followed by stain differentiation with 1% v/v acid alcohol (80% ethanol, 19% deionized water, 1% HCl), 0.3% v/v ammonia water (0.3% NH_4_OH in de-ionized H_2_0) and washing with 70% ethanol. After eosin staining (0.05% Eosin Y in 70% Ethanol-0.005% acetic acid), sections were dehydrated with increasing serial dilutions of ethanol and xylene. Slides were mounted using permount mounting medium. *n* = 5 representative tumors for control group and *n* = 4 representative tumors for Andrographolide 10 mg/kg group.

### Immunohistochemistry and immunofluorescence *in vivo*

Paraffin-embedded tumor tissue sections were dewaxed in xylene and rehydrated using a descending concentration of alcohol. Antigen retrieval was performed using a citrate-based Antigen Unmasking Solution (Vector Laboratories Burlingame, Ca, USA). Endogenous peroxidase was quenched with 3% v/v H_2_O_2._ Sections were blocked (10% FBS) for 1 hour followed by an overnight incubation with a primary antibody. The primary antibodies used were CD-31 (1:50), MMP11 (1:500), pH (1:1000) (Abcam, Cambridge; MA, USA) and Ki-67 (1:50) (Vector Laboratories; Burlingame, CA, USA). For immunofluorescence, the secondary antibody used was Alexa-Fluor 594 (anti-rabbit) 1:2000 (Molecular Probes, Life Technologies, Carlsbad, CA, USA) and nuclei were stained with DAPI 1:5000 (Santa Cruz Biotechnology, Santa Cruz, CA, USA). All immunohistochemical sections were detected using Dako Envision system-HRP (DAB) (anti-rabbit) (Dako; Glostrup, Denmark) according to the manufacturer's instructions. Digital photographs were obtained using an Olympus IX71 Inverted microscope (Olympus America, Melville, NY).

### Microarray analysis

Affymetrix gene chip based transcript profiling was carried out at the RCMI Center for Genomics in Health Disparities and Rare Diseases (University of Puerto Rico, Medical Sciences Campus). RNA was isolated from tumor tissue using RNeasy Mini Kit (Qiagen Inc., Valencia; CA, USA). Total RNA (100 ng) was converted to cDNA and amplified using T7 oligo dT and the GeneChip^®^ WT cDNA Synthesis Kit, the GeneChip^®^ WT cDNA Amplification Kit, and the GeneChip^®^ Sample Cleanup Module exactly as described in the GeneChip^®^ Whole Transcript (WT) Sense Target Labeling Assay Manual Addendum. Quality control steps were performed to ensure that the RNA was adequate to be used in the first strand cDNA synthesis (10 μg of RNA). A gel-shift analysis of the WT (Whole Transcript) was done to assess the labeling efficiency of the fragmented cDNAs. The image data was normalized using the Expression Console software provided by Affymetrix. Gene expression values and clustering was done using the Transcriptome Analysis Console also provided by Affymetrix. The settings used to identify differences in expression were: a fold change higher than 2 or lower than –2 and a *p* value lower than 0.05. Identification of gene expression patterns was done with Ingenuity Pathway Analysis (IPA) software and parameters used were: a fold change higher than 1.5 or lower than –1.5 and a *p* value lower than 0.05. To identify the affected functions and networks we used the “diseases and functions” sections of the IPA software. Data was documented according to the MIAME guidelines [62].

### Real time PCR validation

To validate microarray results, quantitative Real time PCR (qRT-PCR) was performed under standard conditions using the Step One Plus Real-time PCR System (Applied Biosystems, Carlsbad; CA, USA). Primers were designed using the Integrated DNA Technologies (IDT) Primer Quest tool. Primers used were: ATM serine/threonine kinase (ATM), Bloom syndrome, RecQ helicase like (BLM), Breast cancer 2, early onset (BRCA2), BRCA1 interacting protein C-terminal helicase 1(BRIP1), Claspin (CLSPN) Nibrin (NBN), and partner and localizer of BRCA2 (PALB2) (Table 2). A BLAST was run to ensure specificity of the sequences. qRT-PCR was performed in 20 μL reactions using SYBR super mix (Bio-Rad, Hercules, CA, USA). The cycle used was, 95° C for 15 seconds and 62° C for 1 minute or 95° C for 15 seconds and 56° C for 1 minute. PCR efficiency was examined and the melting curve data was collected for PCR specificity. The housekeeping gene used was GAPDH. Quantification was done using the ΔΔC_t_ method. No PCR product was detected in control samples in which the template was omitted. Statistical analysis was done with a Mann–Whitney *U* test at a 95% confidence interval. A total of three tumors per group were used. Experiments were performed in triplicates.

### Immunofluorescence detection of histone H2AX phosphorylation

22RV1, PC3 and RWPE1 cells were treated with DMSO (control) or Andrographolide at 25 μM for 24 hours. Cells were fixed, permeabilized, blocked with FBS (10%) and stained with DAPI, to visualize nucleus, and phospho-H2AX (1:400) to measure the amount of phospho-H2AX formed. The secondary antibody used was Alexa-Fluor 594. Digital photographs were obtained using an Olympus IX71 Inverted microscope (Olympus America, Melville, NY). Image Pro Plus was used to determine a ratio of the formation of phospho-H2AX and total cells. The experiment was done in triplicates and data was analyzed using GraphPad Prism Student *t*-test at 95% confidence interval.

### Detection of DNA damage

DNA Damage assay was performed by flow cytometry using the MUSE Multicolor DNA Damage Kit, following manufacturer's instructions. PC3 cells were treated with DMSO (control) or Andrographolide (25 μM) for 24 hours. The experiment was performed using 18 samples, in triplicates and data was analyzed using GraphPad Prism Student *t*-test at 95% confidence interval.

### Statistical analysis

Statistical analysis was performed using the GraphPad PRISM software. Results represent the mean ± standard error of the mean (SEM). Differences between groups were analyzed using the Student's *t*-test or one-way ANOVA followed by Dunnett's test. *P* < 0.05 was considered statistically significant.

## SUPPLEMENTARY MATERIALS FIGURES



## Abbreviations

PCa: prostate cancer
MMP11: matrix metalloproteinase 11
ATM: ATM serine/threonine kinase
BLM: Bloom syndrome, RecQ helicase like
BRCA2: Breast cancer 2, early onset
BRIP1: BRCA1 interacting protein C: terminal helicase 1
CLSPN: claspin
NBN: nibrin
PALB2: partner and localizer of BRCA2
DSB: DNA double-strand breaks
γH2AX: phosphorylated H2AX
DDR: DNA Damage Respone
FBS: fetal bovine serum
BPE: bovine pituitary extract
DMSO: dimethyl sulfoxide
qRT-PCR: Real time PCR
IPA: Ingenuity Pathway Analysis
SEM: Standard error of the mean.
