# Supplementary material for: Andrographolide induces DNA damage in prostate cancer cells
Source: Oncotarget. 2019 Feb 1;10(10):1085–101. doi: 10.18632/oncotarget.26628 (PMC6383681; doi:10.18632/oncotarget.26628)
Supplement: Supplementary file 1 [file oncotarget-10-1085-s001.pdf]

Andrographolide induces DNA damage in prostate cancer cells

SUPPLEMENTARY MATERIALS

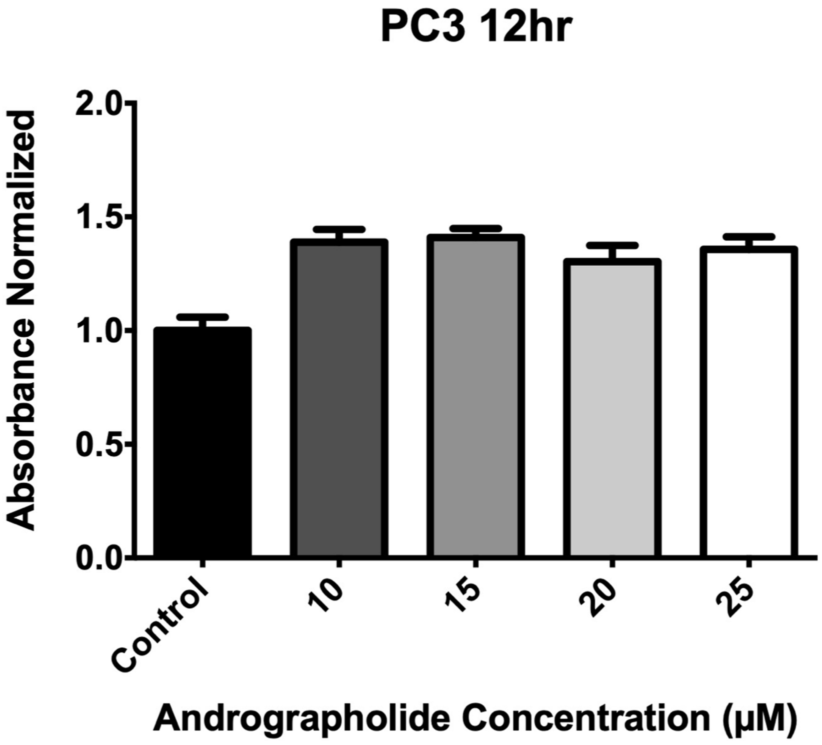

Supplementary Figure 1: Andrographolide decreased cell viability in prostate cancer cells. PC3 cells were treated with Andrographolide for 12 hours, but there were no significant changes in proliferation.

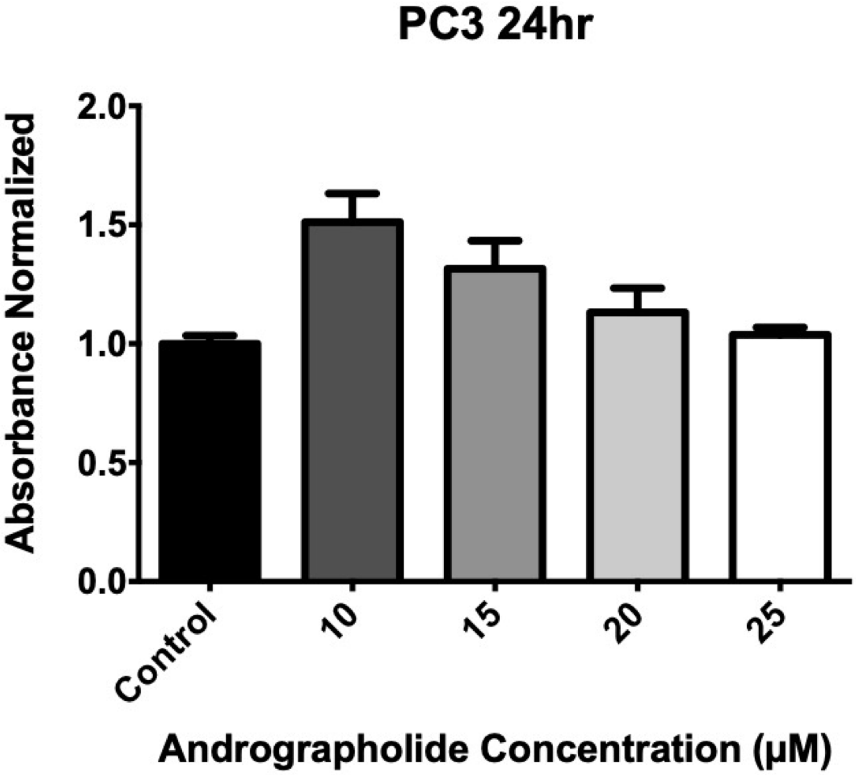

Supplementary Figure 2: Andrographolide decreased cell viability in prostate cancer cells. PC3 cells were treated with Andrographolide for 24 hours, but there were no significant changes in proliferation.

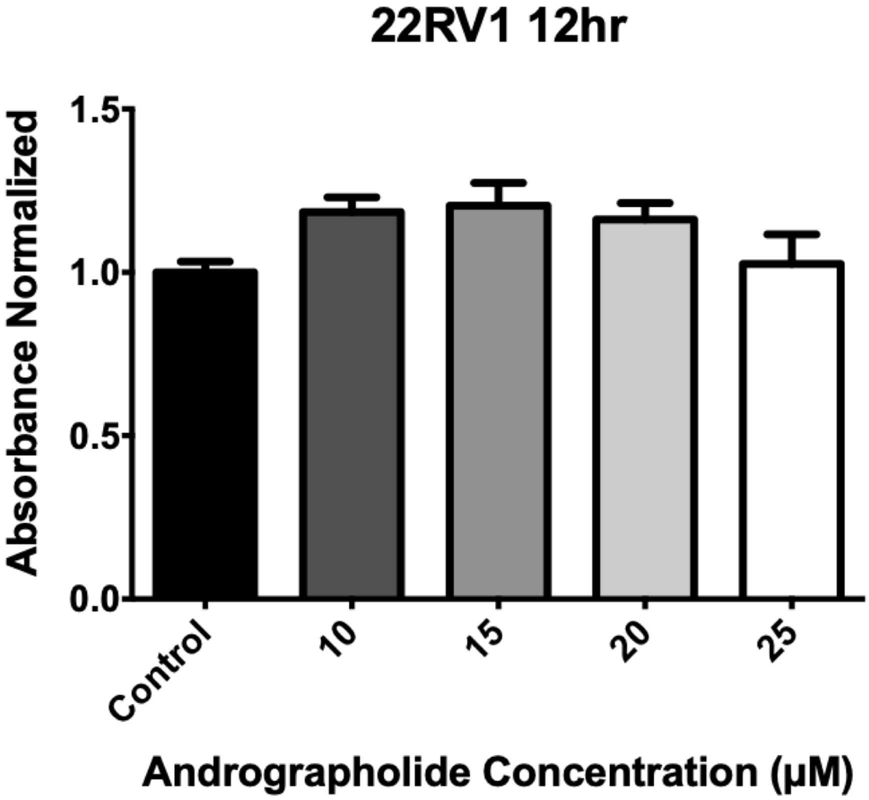

Supplementary Figure 3: Andrographolide decreased cell viability in prostate cancer cells. 22RV1 cells were treated with Andrographolide for 12 hours, but no significant changes were observed in the proliferation of these cells.

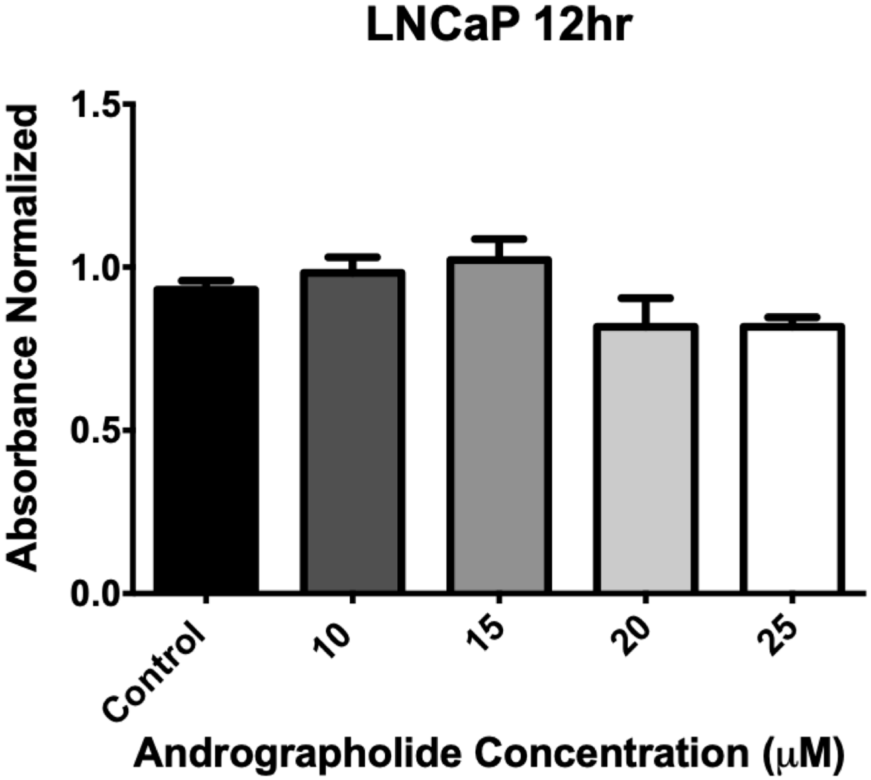

Supplementary Figure 4: Andrographolide decreased cell viability in prostate cancer cells. LNCaP cells were treated with Andrographolide for 12 hours, but there were no significant changes in proliferation.

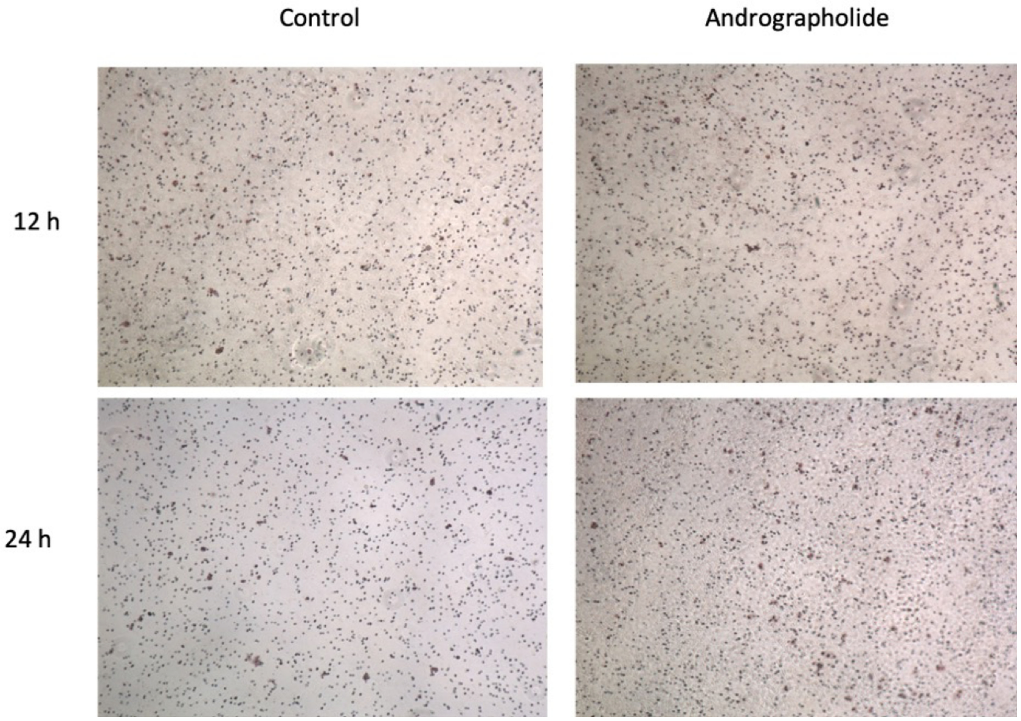

Supplementary Figure 5: Andrographolide did not change invasion in 22RV1 cells. The effect of Andrographolide (25 μM) in invasion was evaluated using transwell chamber method. 22RV1 cells were treated for 12 and 24 hours. Andrographolide did not change invasion in androgen dependent cells (22RV1).
